# Supplementary material for: Effectiveness and Safety of Ayurvedic Medicines in Type 2 Diabetes Mellitus Management: A Systematic Review and Meta-Analysis
Source: Front Pharmacol. 2022 Jun 8;13:821810. doi: 10.3389/fphar.2022.821810 (PMC9213670; doi:10.3389/fphar.2022.821810)
Supplement: Supplementary file 1 [file DataSheet1.zip › Supplementary Material/Forest and Funnel Plots/Nigella sativa/FBG.pdf]

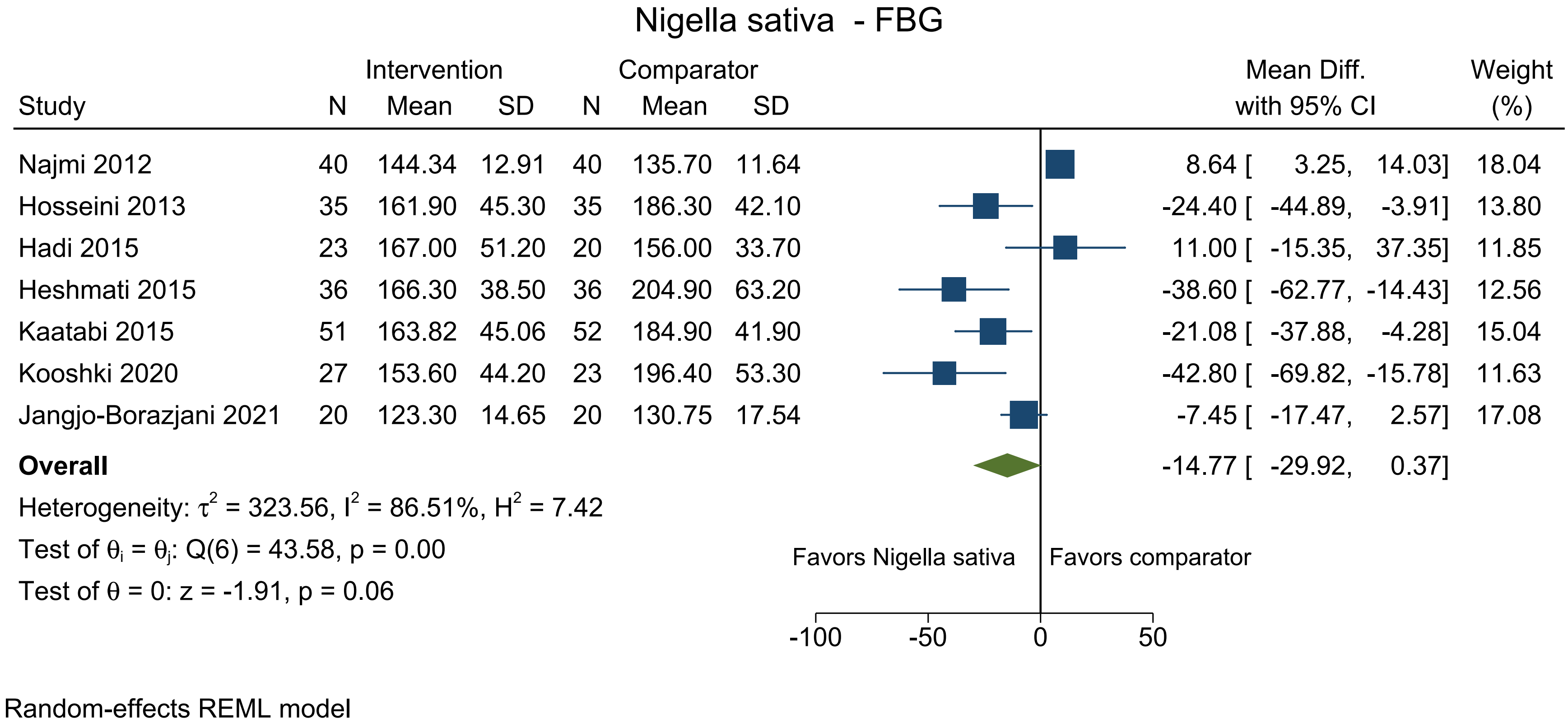
Nigella sativa - FBG
Intervention
Comparator
Mean Diff. with 95% CI
Weight (%)
Study
N
Mean
SD
N
Mean
SD
Najmi 2012 40 144.34 12.91 40 135.70 11.64 8.64 [3.25, 14.03] 18.04
Hosseini 2013 35 161.90 45.30 35 186.30 42.10 -24.40 [-44.89, -3.91] 13.80
Hadi 2015 23 167.00 51.20 20 156.00 33.70 11.00 [-15.35, 37.35] 11.85
Heshmati 2015 36 166.30 38.50 36 204.90 63.20 -38.60 [-62.77, -14.43] 12.56
Kaatabi 2015 51 163.82 45.06 52 184.90 41.90 -21.08 [-37.88, -4.28] 15.04
Kooshki 2020 27 153.60 44.20 23 196.40 53.30 -42.80 [-69.82, -15.78] 11.63
Jangjo-Borazjani 2021 20 123.30 14.65 20 130.75 17.54 -7.45 [-17.47, 2.57] 17.08
Overall -14.77 [-29.92, 0.37]
Heterogeneity: τ^2 = 323.56, I^2 = 86.51%, H^2 = 7.42
Test of $\theta_i = \theta_j$: Q(6) = 43.58, p = 0.00
Test of θ = 0: z = -1.91, p = 0.06
Favors Nigella sativa
Favors comparator
-100
-50
0
50
Random-effects REML model
